# Detecting amyloid positivity from MRI scans using comprehensible convolutional neural networks

**DOI:** 10.1002/alz.089044

**Published:** 2025-01-09

**Authors:** Fedor Levin, Qingyue Li, Devesh Singh, Katharina Buerger, Klaus Fliessbach, Michael T. Heneka, Luca Kleineidam, Christoph Laske, Robert Perneczky, Oliver Peters, Josef Priller, Alfredo Ramirez, Anja Schneider, Annika Spottke, Michael Wagner, Jens Wiltfang, Emrah Düzel, Frank Jessen, Stefan Teipel, Martin Dyrba, the DELCODE Study Group

**Affiliations:** ^1^ German Center for Neurodegenerative Diseases (DZNE), Rostock Germany; ^2^ Rostock University Medical Center, Rostock Germany; ^3^ German Center for Neurodegenerative Diseases (DZNE), Munich Germany; ^4^ Institute for Stroke and Dementia Research (ISD), University Hospital, LMU, Munich Germany; ^5^ German Center for Neurodegenerative Diseases (DZNE), Bonn Germany; ^6^ University of Bonn Medical Center, Dept. of Neurodegenerative Disease and Geriatric Psychiatry/Psychiatry, Bonn Germany; ^7^ Luxembourg Centre for Systems Biomedicine (LCSB), University of Luxembourg, Luxembourg Luxembourg; ^8^ German Center for Neurodegenerative Diseases (DZNE), Tuebingen Germany; ^9^ Section for Dementia Research, Hertie Institute for Clinical Brain Research and Department of Psychiatry and Psychotherapy, University of Tuebingen, Tuebingen Germany; ^10^ LMU University Hospital, Munich Germany; ^11^ Munich Cluster for Systems Neurology (SyNergy), Munich Germany; ^12^ Department of Psychiatry and Psychotherapy, University Hospital, LMU Munich, Munich Germany; ^13^ Charité – Universitätsmedizin Berlin, corporate member of Freie Universität Berlin and Humboldt‐Universität zu Berlin – Institute of Psychiatry and Psychotherapy, Berlin Germany; ^14^ German Center for Neurodegenerative Diseases (DZNE), Berlin Germany; ^15^ School of Medicine, Technical University of Munich; Department of Psychiatry and Psychotherapy, Munich Germany; ^16^ University of Edinburgh and UK DRI, Edinburgh UK; ^17^ Department of Psychiatry and Psychotherapy, Charité, Berlin Germany; ^18^ Division of Neurogenetics and Molecular Psychiatry, Department of Psychiatry and Psychotherapy, Faculty of Medicine and University Hospital Cologne, University of Cologne, Cologne Germany; ^19^ Department of Psychiatry & Glenn Biggs Institute for Alzheimer’s and Neurodegenerative Diseases, San Antonio, TX USA; ^20^ Excellence Cluster on Cellular Stress Responses in Aging‐Associated Diseases (CECAD), University of Cologne, Cologne Germany; ^21^ Department of Neurology, University of Bonn, Bonn Germany; ^22^ German Center for Neurodegenerative Diseases (DZNE), Goettingen Germany; ^23^ Department of Psychiatry and Psychotherapy, University Medical Center, University of Goettingen, Goettingen Germany; ^24^ Neurosciences and Signaling Group, Institute of Biomedicine (iBiMED), Department of Medical Sciences, University of Aveiro, Aveiro Portugal; ^25^ German Center for Neurodegenerative Diseases (DZNE), Magdeburg Germany; ^26^ Institute of Cognitive Neurology and Dementia Research (IKND), Otto‐von‐Guericke University, Magdeburg Germany; ^27^ Department of Psychiatry, University of Cologne, Medical Faculty, Cologne Germany; ^28^ Department of Psychosomatic Medicine, Rostock University Medical Center, Rostock Germany

## Abstract

**Background:**

Analysis of neuroimaging data based on convolutional neural networks (CNNs) can improve detection of clinically relevant characteristics of patients with Alzheimer’s disease (AD). Previously, our group developed a CNN‐based approach for detecting AD via magnetic resonance imaging (MRI) scans and for identifying features that are relevant to the decision of the network. In the current study, we aimed to evaluate the potential utility of applying this approach to MRI scans to assist in the identification of individuals at high risk for amyloid positivity to aid in the selection of study samples and case finding for treatment.

**Method:**

In the current analysis, we have trained a CNN to detect amyloid positivity using MRI scans from 1461 Alzheimer’s Disease Neuroimaging Initiative (ADNI) participants (498 cognitively normal participants, 103 participants with significant memory concern, 640 participants with mild cognitive impairment, and 220 participants with AD dementia). Amyloid positivity was assessed via amyloid PET scans obtained with [^18^F]florbetapir or [^18^F]florbetaben and quantified on a Centiloid scale. A threshold of 24.1 CL categorized 46% of participants as amyloid‐positive. The modeling approach was evaluated using 10‐fold cross‐validation, the number of epochs in training was set to 10.

**Result:**

For each of 10 cross‐validation folds, we selected a model state corresponding to an epoch showing best performance in the validation partition. Balanced accuracy across these models ranged from 0.62 to 0.72 with an average of 0.68 (SD = 0.03).

**Conclusion:**

We used a previously established approach to train CNNs for detecting amyloid positivity using MRI scans. Such models, particularly when tuned to have low rates of false negatives, have a potential to enhance identification of patients who would benefit from more in‐depth assessments, which could then inform antibody treatment. We are conducting ongoing work to improve and characterize the modeling approach, including evaluation of relevance maps which indicate importance of brain regions for detecting amyloid positivity. Future work will evaluate the role of amyloid positivity threshold selection. Planned analyses also include validation in independent data such as the German DZNE ‐ Longitudinal Cognitive Impairment and Dementia Study (DELCODE) dataset.

